# Sex differences in adipose insulin resistance are linked to obesity, lipolysis and insulin receptor substrate 1

**DOI:** 10.1038/s41366-024-01501-x

**Published:** 2024-03-15

**Authors:** Peter Arner, Nathalie Viguerie, Lucas Massier, Mikael Rydén, Arne Astrup, Ellen Blaak, Dominique Langin, Daniel Peter Andersson

**Affiliations:** 1 Department of Medicine H7, Karolinska Institutet, Stockholm, Sweden; 2 Institute of Metabolic and Cardiovascular Diseases, I2MC, University of Toulouse, Inserm, Toulouse III University - Paul Sabatier (UPS), Toulouse, France; 3 Department of Endocrinology and Metabolism, Karolinska University Hospital, Stockholm, Sweden; 4 Department of Obesity and Nutritional Sciences, Novo Nordisk Foundation, 2900 Hellerup, Denmark; 5 Department of Human Biology, NUTRIM, School of Nutrition and Translational Research in Metabolism, Faculty of Health, Medicine and Life Science, Maastricht University, 6200 MD Maastricht, The Netherlands; 6 Centre Hospitalier Universitaire de Toulouse, Toulouse, France; 7 Institut Universitaire de France (IUF), Paris, France

**Keywords:** Translational research, Obesity, Type 2 diabetes

## Abstract

**Background/Objective:**

Insulin resistance is more prominent in men than women. If this involves adipose tissue is unknown and was presently examined.

**Subjects/Methods:**

AdipoIR (in vivo adipose insulin resistance index) was measured in 2344 women and 787 men. In 259 of the women and 54 of the men, insulin induced inhibition of lipolysis (acylglycerol breakdown) and stimulation of lipogenesis (glucose conversion to acylglycerols) were determined in subcutaneous adipocytes; in addition, basal (spontaneous) lipolysis was also determined in the fat cells. In 234 women and 115 men, RNAseq expression of canonical insulin signal genes were measured in subcutaneous adipose tissue. Messenger RNA transcripts of the most discriminant genes were quantified in 175 women and 109 men.

**Results:**

Men had higher AdipoIR values than women but only when obesity (body mass index 30 kg/m^2^ or more) was present (*p* < 0.0001). The latter sex dimorphism was found among physically active and sedentary people, in those with and without cardiometabolic disease and in people using nicotine or not (*p* = 0.0003 or less). In obesity, adipocyte insulin sensitivity (half maximum effective hormone concentration) and maximal antilipolytic effect were tenfold and 10% lower, respectively, in men than women (*p* = 0.005 or less). Basal rate of lipolysis was two times higher in men than women (*p* > 0.0001). Sensitivity and maximum effect of insulin on lipogenesis were similar in both sexes (*p* = 0.26 and p = 0.18, respectively). When corrected for multiple comparison only RNAseq expression of insulin receptor substrate 1 (*IRS1*) was lower in men than women (*p* < 0.0001). The mRNA transcript for *IRS1* was 60% higher in women than men (*p* < 0.0001).

**Conclusions:**

In obesity, adipose tissue insulin resistance is more pronounced in men than in women. The mechanism involves less efficient insulin-mediated inhibition of adipocyte lipolysis, increased basal rate of lipolysis and decreased adipose expression of a key element of insulin signaling, *IRS1*.

## Introduction

There is now ample evidence that men are more prone than women to develop type 2 diabetes mellitus (T2DM) at younger age and lower degrees of fat mass [1]. Many factors may explain this sexual dimorphism but differences between men and women in insulin action could be of great importance because insulin resistance is paramount for the development of T2DM [2]. Indeed, it is frequently observed that skeletal muscle and liver are more sensitive to insulin in women than men [3–5]. If adipose tissue also is involved in the sex differences is unknown and was presently examined. This organ plays a specific role for insulin regulation of energy homeostasis due to the action of the hormone on fatty acid metabolism. These lipids are stored as triglycerides in fat cells and less efficient ability of insulin to inhibit breakdown (antilipolysis) and/or stimulate synthesis of the triglycerides could elevate the circulating fatty acid levels and, in turn, cause insulin resistance [6, 7]. Thus, sex dimorphism in insulin action on adipose lipid metabolism could have a different impact on T2DM pathogenesis than insulin resistance of glucose metabolism in liver and muscle.

Adipose insulin action is usually determined with cumbersome and resource demanding methods, which are not suitable for large scale studies [8]. More recently, a simple indirect technique was introduced which is based on the product of circulating concentrations of fasting fatty acids (mmol/l) and insulin (pmol/l), termed AdipoIR [9]. This index correlates strongly with measures of insulin action in vivo [10] and in fat cells in vitro [11]. Herein we investigated AdipoIR in adult subjects to elucidate possible sex differences. Because obesity has a very strong effect on insulin action [12] we subdivided the participants according to body mass index (BMI) of 30 kg/m^2^ as cut-off value for obesity.

To elucidate cellular mechanisms, we also studied basal (spontaneous) rate of lipolysis and the action of insulin on lipolysis and lipogenesis in isolated subcutaneous fat cells in a subgroup. These measures reflect triglyceride breakdown and synthesis from glucose, respectively, in adipose tissue. The metabolic studies suggested a role of initial steps in insulin signaling for sex differences of hormone action in adipose tissue. This was further explored in subcutaneous adipose tissue by measuring the mRNA expression of genes involved in the canonical insulin signal pathway; this cascade regulates insulin action on fat cell metabolism [13]. Several hitherto unknown factors related to sex differences in adipose insulin resistance were revealed with potential clinical importance for the development of T2DM in obesity.

## Methods

### Subjects

From 1993 to 2020, one of the present investigators (PA) enrolled 6647 subjects living in the Stockholm area, Sweden, for different studies related to adipose tissue function, such as clinical findings, fat cell metabolism/endocrinology and genetic studies as exemplified [11, 14, 15]. Herein we included all adult subjects having data for AdipoIR, namely 2343 women and 787 men. The cohort is termed KAROLINSKA. They were recruited by local advertising and self-reported to be in general good health. About 5% were of non-European origin. Patients with type 1 diabetes were excluded because they were not standardized for insulin treatment. We also excluded subjects with acute severe diseases as it was considered unethical to let them undergo invasive studies and they would not be representative for the other subjects. The participants came to the laboratory in the overnight fasting state at 08 a.m. and underwent clinical investigations by the same three research nurses during the study period. All subjects were body weight stable for at least 3 months according to self-report. Abdominal subcutaneous adipose tissue was obtained by needle aspiration from those with obesity (BMI, 30 kg/m^2^ or more) throughout the course of the study, when the removed amounts enabled analysis of insulin action (see below). Height, body weight, and body fat were determined followed by venous blood sampling for routine clinical chemistry measures [14]. Fatty acid and insulin values were used to calculate in vivo adipose tissue insulin resistance (AdipoIR) [11] which indirectly reflects insulin action on lipolysis and lipogenesis in fat cells [11]. Glucose and insulin values were used to calculate another index of insulin resistance (HOMA-IR) as described [11]. Physical activity was assessed by a four-graded scale where 1 was almost completely sedentary and 4 was strong physical activity for > 30 min at least 5 times/week as described [15]. These scores have been validated and are highly specific for classification into a sedentary (score 1) or active (score 2 or more) phenotype [15]. A second group of adult subjects with obesity, termed DiOGenes, were used solely for gene expression analysis in subcutaneous adipose tissue. They participated in the DiOGenes study [16], which is a pan-European, multi-center, randomized controlled dietary intervention program (NCT00390637). Herein we investigated 115 men and 234 women with obesity having data on abdominal subcutaneous adipose gene expression by RNAseq (see below) using the results from the baseline examination. In 179 women and 109 men from DiOGenes gene expression data was confirmed by quantitative real time polymerase chain reaction (RT-qPCR). Clinical data were collected in the same way as for KAROLINSKA and described before [16]. The KAROLINSKA data collection is based on data from several previous projects, and all have been approved by the Regional Ethics Committee in Stockholm, Sweden (Diary numbers 114/92, 200/98, 117/99, 167/02, 592/03, 534/03, 163/03, 2008/1010-31/3, 2011/1102 31/1, 2016/2583 and 31/1, 2018/809-31). The ethics permit from 2018 allowed us to retrospectively analyze all clinical and adipose data from these previously approved applications. The different studies used in KAROLINSKA were explained in detail by the investigators to each participant and informed written consent was obtained. DiOGenes (NCT00390637) studies were performed according to the latest version of the Declaration of Helsinki. Local ethics committees at the different investigation sites in Europe approved all procedures and written informed consent was obtained from all participants.

### Adipose tissue examinations

The procedures for KAROLINSKA are as follows and were performed by the same four laboratory technicians. Collagenase isolated fat cells were prepared and used for lipolysis and lipogenesis as described [17]. For lipolysis, diluted fat cell suspensions were incubated in duplicate in the absence of any lipolysis acting agent (basal) and with or without insulin (0–70 nmol/l) for 2 h at 37 ^o^C and in buffers (pH 7.4) containing glucose, albumin, adenosine deaminase (to remove adenosine which inhibits lipolysis) and 1 mmol/l of 8-bromo-cyclic-AMP. The latter synthetic nucleotide is an excellent tool for measuring the antilipolytic effect of insulin in human fat cells [18]. Glycerol in the medium was measured as indicator of lipolysis [19]. For lipogenesis fat cells were incubated for 2 h at 37 ^o^C in duplicate in a 2% (vol/vol) buffer (pH 7.4) containing glucose (1 µmol/l), albumin and tracer amounts of 3-^3^H glucose with or without varying insulin concentrations (0–70 nmol/l). After incubation, radioactivity in the total incubate was determined and used for calculating the amount of glucose incorporated into fat cell lipids. This lipogenesis method has been evaluated in detail [20]. It measures the incorporation of radioactive glucose carbons into the glycerol and fatty acid moieties of the fat cell lipid droplet. There is no consensus on how to express absolute rates of lipolysis/lipogenesis. Herein, we expressed basal lipolysis as glycerol release per lipid weight or number of fat cells and the insulin action as relative values using the ratio: presence of insulin divided by no insulin in the incubation medium. Responsiveness was defined as the ratio at the maximum effective insulin concentration. Insulin sensitivity was determined by measuring the half maximum effective hormone concentration from the concentration-response curves. This value was transformed to the negative 10 log molar value (pD2). Responsiveness and pD2 reflect receptor distal and near events, respectively, for hormones acting through spare receptors [21] which is the case for insulin [22]. We could not always make complete lipolysis/lipogenesis experiments. In such a case we prioritized lipogenesis. The studies of DiOGenes were conducted as follows. For RNAseq and RT-qPCR total RNA was extracted, quantified and quality checked as described [23]. Gene expression was then examined by using 100-nucleotide long paired-endRNA sequencing with an Illumina HiSeq 2000 of libraries prepared by using the Illumina TruSeq kit following the manufacturer’s standard protocols. Sequencing was performed for samples having both baseline and after-treatment investigation but herein only the former samples were used. Demultiplexing was carried out with Casava [24]; the resulting FASTQ files were then mapped onto the human genome (GRCh37 assembly) with RNA-STAR [25] with the use of default parameters. Sequencing quality was evaluated by using FastQC [26]. Mapping quality was assessed by using Rsamtools [27]. The number of reads mapping onto genes was retrieved by using GenomicAlignments [28]. Annotation was performed by using 64,102 genes from the GRCh37.75 assembly generated with the use of the AnnotationDbi R package [29]. The values for mRNA are presented as log 2 transformed relative expression. In the present study we selected from the array 17 expressed genes regulating the early steps of insulin signaling in the canonical pathway [19] because the pharmacological studies of lipolysis/lipogenesis suggested involvement of these events. For the validation we investigated insulin receptor substates 1 and 2 (*IRS1* and *2*) by quantitative real time polymerase chain reaction (RT-qPCR) because data were available from a previous study where the methods are described in detail [23]. In brief, complementary DNA was prepared from total RNA and processed by using the BioMark HD system with 96.96 Dynamic array IFC (BioMark) and Taqman assays with commercial Taqman probes (Applied Biosystems), Hs00178563_m1 for *IRS1* and Hs00275843_s1 for *IRS2*, according to the protocol described by Viguerie et al. [30]. *IRS1* and 2 expression was related to expression of the house keeping mRNA *GUSB* (glucuronidase beta, Hs00939627_m1). There was no sex effect on the expression of *GUSB*.

### Statistics

Analyses were performed in JMP Version 16.1.0 (SAS, Institute Inc., Buckinghamshire, UK). Values for several insulin action parameters in fat cells (primary endpoints) could not be normalized. Therefore, we presented results as median with 25% quartiles in tables and text or as box plots in figures with 10-90 percentiles and used Wilcoxon’s two sample test to compare two groups of values. When several factors were compared for relation with metabolic effects of insulin, we used analysis of covariance (ANCOVA). Besides sex we included co-factors considered to be important for insulin action. Those were sex, BMI or % body fat, fasting plasma glucose, and age, which were available in both cohorts and were not influenced by each other in an important way. For all comparisons a two-tailed test was used. In the analysis of gene expression a Bonferroni corrected *p* value of <0.00185 (<0.05/27) was used to define a statistically significant difference because we had not a priory hypothesis for which of the 27 genes investigated that were involved in the metabolic differences observed. Otherwise, a *p* value < 0.05 was considered as statistically significant. We also compared % body fat with pD2 for antilipolysis using Spearman correlation followed by an investigation of sex interaction using ANCOVA. Prior to termination of inclusion of KAROLINSKA subjects we made a power calculation using linear methods and previously recorded values for AdipoIR [15]. In two groups of equal size, we could detect a 0.5 difference in AdipoIR between the sexes in 50 subjects of each group with 80% power and at *p* = 0.05 using two-sided t-test. As the women group was much larger the statistical power calculation suggests that we had adequate statistical power in the present study to study small subgroups.

## Results

The clinical data with the two groups are shown in Table S1. As expected, and regardless of obesity was present or not, men displayed a less favorable metabolic profile, including higher HOMA-IR values, than women although the women had more body fat than men. In general men were also slightly older than the women.

**Fig. 1:**
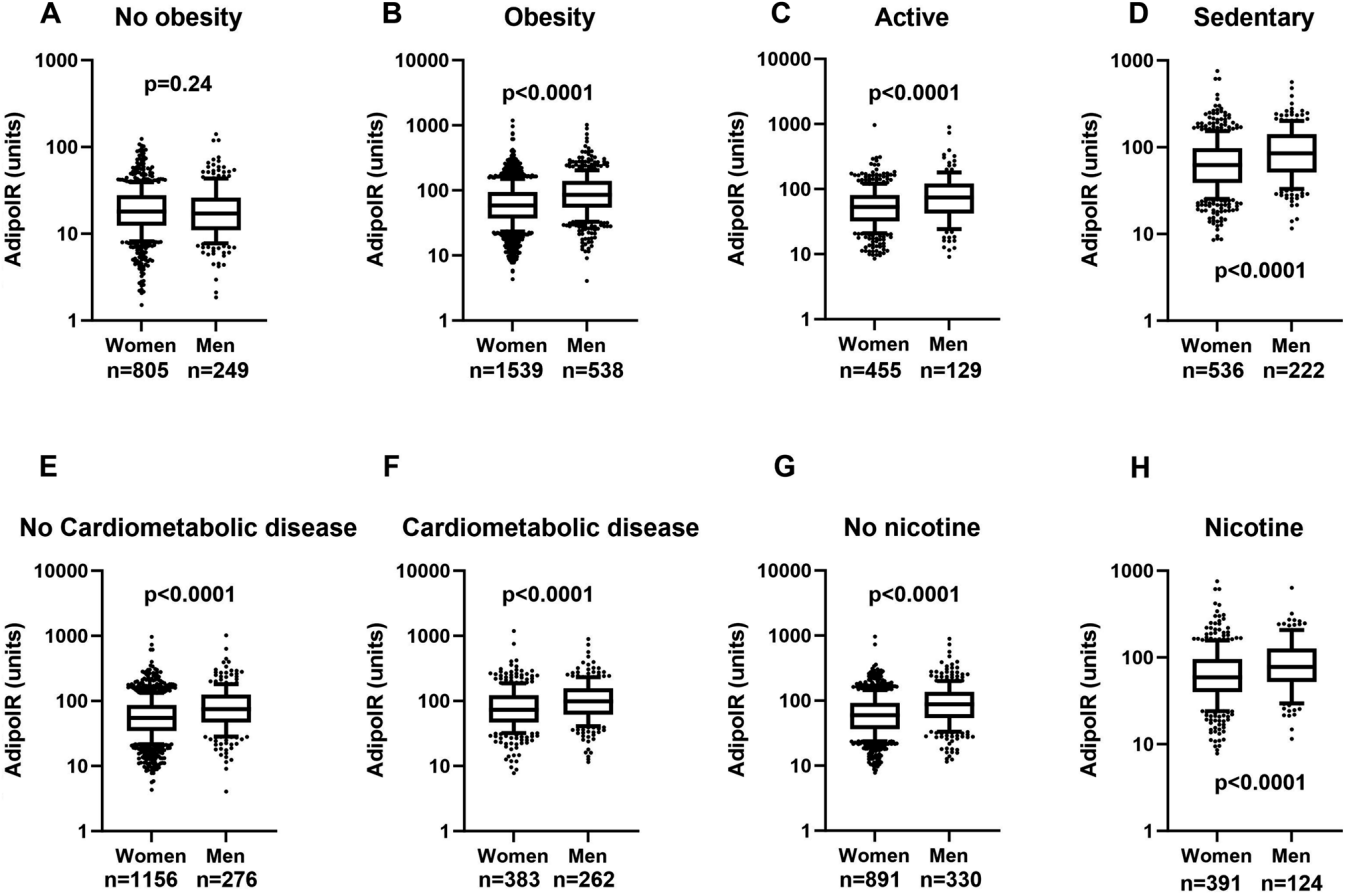
Findings with AdipoIR (10-log scale of pmol/l of fasting insulin times mmol/l of fasting fatty acids). First those without (**A**) or with (**B**) obesity were compared for sex differences. Thereafter subgroups of subjects with obesity were compared. **C** active. **D** sedentary. **E** no cardiometabolic disease (CMD). **F** having CMD. **G** no nicotine use. **H** nicotine use. Values are box plots. Wilcoxon’s two sample test was used. CMD is defined as having diagnosed type 2 diabetes, hypertension, hyperlipidemia, or cardiovascular disease. n=number of subjects.

Results with AdipoIR in the KAROLINSKA group are shown in Fig. 1. These measures were obtained from a single center using the same method for determining circulating insulin and fatty acids. There was no influence of sex in those without obesity (Fig. 1A). However, when comparing men and women with obesity, (Fig. 1B) men had higher AdipoIR values than women (*p* < 0.0001). Because of the lack of sex difference in AdipoIR among subjects without obesity all subsequent studies were focused on subjects living with obesity. AdipoIR was also measured in a subgroup of the DiOGenes study. However, the underlying insulin and fatty acid values were subject to strong site variations according to analysis of variance (F = 2.5 and 6.4, respectively; *p* = 0.015 and <0.0001, respectively), most probably due to the fact that DIOGENES includes subjects from eight different European investigation centers using different analyses pipelines. Consequently, the AdipoIR measures in DiOGenes were not used in this study. Furthermore, HOMA-IR and AdipoIR were not compared because both measures use insulin as factor in the calculation of insulin resistance values.

Several obesity subgroup analyses of AdipoIR were performed in the KAROLINSKA cohort (Fig. 1C–H). Higher AdipoIR values among men than women were recorded in both physically active and sedentary people, in those with and without concomitant metabolic disorder (T2DM, hypertension, hyperlipidemia or cardiovascular disease) and using nicotine or not (*p* = 0.0003 or less).

**Fig. 2:**
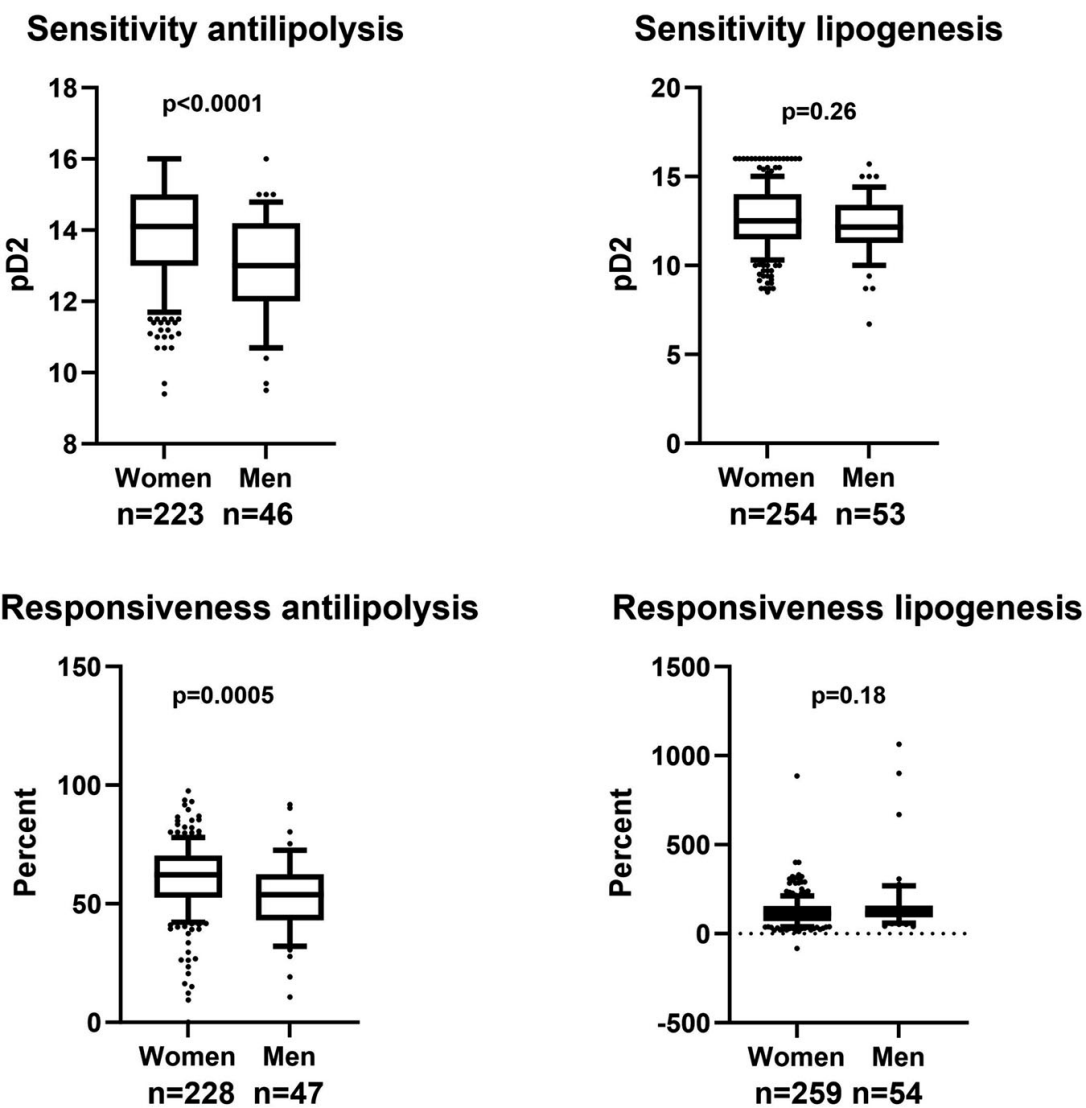
Effect of insulin on metabolism in subjects with obesity. Isolated fat cells were incubated without or with insulin in different concentrations. The sensitivity and responsiveness of hormone induced inhibition of lipolysis (antilipolysis) and stimulation of lipogenesis were investigated. Sensitivity is half maximum effective concentration expressed as pD2. Responsiveness is % maximum effect. Values are compared by Wilcoxon’s two sample test. n= number of subjects.

Next, insulin action on lipolysis or lipogenesis was examined in subjects with obesity (Fig. 2). Two pharmacological aspects could be analyzed from the concentration-response experiments, namely pD2 which reflects insulin sensitivity and thereby proximal receptor signal events and maximum action (responsiveness) which mirrors more distal actions of insulin on lipolysis and lipogenesis, respectively. For lipogenesis, neither the insulin sensitivity nor the insulin responsiveness was subjected to sexual dimorphism (*p* = 0.26 and *p* = 0.18, respectively). However, antilipolysis was subjected to clear sex difference. Values for pD2 were about one log unit lower in men than in women (*p* < 0.0001) which corresponds to a 10-fold lower higher half maximum effective concentration in men. In addition, maximum inhibition of lipolysis was around 10% lower in men than women (*p* = 0.0005).

The sex differences in pD2 for antilipolysis were further examined by correlating these values with % body fat (Fig. S1). There was a weak (Rho = 0.32) but significant (*p* < 0.0001) positive correlation between the values in the whole study group with obesity. Furthermore, the relationship was influenced by sex (F = 5.2; *p* = 0.023).

The pharmacological data in Fig. 2 suggested that variations in insulin signal steps in the canonical pathway for hormone action could be related to the observed sex differences in lipolysis inhibition. To examine this notion, we analyzed mRNA data of subcutaneous adipose tissue in the DiOGenes cohort, which only included persons with obesity (Table 1). These subjects displayed similar sex differences in clinical characteristics as those with obesity in KAROLINSKA (Table S1). We first analyzed the expression of genes encoding proteins in the canonical pathway with RNAseq and focused on the 17 ones we considered to be most important. In addition, we investigated another 10 genes related to basal lipolysis (see below). Because we had no *à priori* hypothesis regarding which of the analyzed genes that are involved we used a Bonferroni correction of the *p* values (*p* < 0.05/27 was defined as statistically significant). Only one gene, *IRS1* encoding insulin receptor substrate 1, was subjected to sex difference with lower expression in men (< 0.0001). In a subset of these people RT-qPCR analysis of *IRS1* and *IRS2* was performed and used for validation (Table 1). The mRNA level of *IRS2* was not subject to a sex influence but *IRS1* expression was 60% higher in women than men (*p* < 0.0001).

**Table 1:** Sex differences in subcutaneous adipose tissue mRNA expression of genes in the canonical insulin signal pathway investigating subjects with obesity.

Gene	RNA seq			RT-qPCR		
	Females (*n* = 234)	Males (*n* = 115)	*p* value	Females (*n* = 175)	Males (*n* = 109)	*p* value
Insulin signaling						
AKT1	9.28 (8.97–9.68)	9.35 (8.95–9.80)	0.32			
AKT2	12.41 (11.91–12.89)	12.24 (11.64–12.61)	0.0022			
INSR	9.997 (9.61–10.23)	10.00 (9.68–10.33)	0.54			
**IRS1**	**8.30 (7.85–8.88)**	**7.93 (7.51–8.53)**	**0.0002**	0.159 (0.114–0.231)	0.100 (0.076–0.131)	<0.0001
IRS2	11.37 (10.77–11.77)	11.14 (10.68–11.65)	0.056	0.624 (0.413–0.967)	0.553 (0.344–0.907)	0.071
PIK3C2A	8.33 (7.32–9.36)	9.03 (7.42–9.95)	0.048			
PIK3C2B	6.61 (6.21–8.12)	7.45 (6.22–8.32)	0.61			
PIK3C2G	3.57 (3.56–3.57)	3.57 (3.33–3.57)	0.11			
PIK3C3	7.25 (6.79–8.41)	8.18 (6.88–8.61)	0.047			
PIK3CB	7.71 (7.38–8.71)	8.33 (7.33–8.72)	0.84			
PIK3CD	7.95 (7.47–8.50)	7.99 (7.59–8.59)	0.17			
PIK3CG	4.93 (4.48–5.89)	5.38 (4.66–6.20)	0.015			
PIK3R1	11.42 (11.10–11.75)	11.45 (11.21–11.75)	0.43			
PIK3R2	5.23 (4.36–5.69)	4.66 (4.14–5.46)	0.0059			
PIK3R3	9.60 (9.19–10.04)	9.73 (9.43–10.21)	0.033			
PIK3R4	6.19 (5.86–7.75)	7.45 (5.85–7.79)	0.49			
PIK3R5	7.11 (5.86-7.75)	7.23 (6.74-7.86)	0.041			

Values are mean and (interquartile range).

They were compared by Wilcoxon’s two sample test. Because 27 genes were investigated with RNAseq in Tables 1, 2 (see “Methods”) and we had no a priori hypothesis which ones that were subjected to sex difference a Bonferroni corrected *p* value was used as being statistically significant, namely <0. 00185 (0.05 / 27). Only one gene fulfilled this criterium (bold style). Two genes were validated with RT-qPCR and statistically compared according to sex as with RNAseq in a subgroup. n= number of subjects.

In the gene expression analyses, adipose tissue was obtained from subjects in several European investigation centers. To study the effect of site interaction we focused on *IRS1* and used ANCOVA. A significant site interaction was observed for RNAseq (F = 6.7, *p* < 0.0001) and RT-qPCR (F = 3.9, *p* = 0.0005). However, in the model the influence of sex on gene expression of *IRS1* was still prominent (F = 15–17 and *p* value 0.0002 or <0.0001). Therefore, we conclude that the dimorphism in *IRS1* expression is not influenced by investigation site differences in any important way.

We also investigated if additional cofactors that by themselves may impact insulin sensitivity would alter the influence of sex on the key findings described above (Table S2). Two ANCOVA models were used. In model one sex, age, fasting glucose, and BMI were included. Sex remained an important contributor to variations in Adipo-IR, pD2 or responsiveness of antilipolysis and *IRS1* gene expression as judged by F-values (10-34, *p* = 0.002 or less). None of the other factors in the model had a consistent and significant influence on the variations in mentioned insulin parameters. In model two, % body fat was substituted for BMI. The results were very similar to those using model one. Thus, the effect of sex was independent of other important cofactors.

Finally, we examined in subjects with obesity possible sex differences in other factors which indirectly may influence insulin action in fat cells (Table 2). Basal rate of lipolysis was two-fold increased in men compared to women (*p* < 0.0001). Among the genes investigated in Table 2 we used the same Bonferroni criterium as for Table 1 (*p* < 0.05/27) to be truly statistically significant. Only the expression of *CIDEA*, *PDE3B*, and receptors for testosterone displayed a sex dimorphism with increased values among men.

**Table 2:** Sex differences in basal lipolysis (expressed as µmoles of glycerol release / 2 h from isolated fat cells) and in adipose gene expression (expressed as 2 log arbitrary units from RNAseq study).

Phenotype	Females (*n* = 234)	Males (*n* = 115)	*p* value
Basal lipolysis			
Per g lipid	0.7 (0.4–1.4)	1.2 (0.7–1.9)	<0.0001
Per 10^7^ fat cells	5.3 (3.2–10.2)	10.0 (5.4–15.2)	<0.0001
mRNA expression			
CIDEA	10.32 (9.80–10.75)	10.60 (10.15–10.94)	0.0002
LIPE	12.59 (12.16–12.87)	12.50 (11.96–12.82)	0.12
PDE3B	10.15 (9.94–10.37)	10.45 (10.27–10.68)	<0.0001
PLIN-1	14.17 (13.98–14.33)	14.01 (13.88–14.16)	0.0047
ATGL	11.77 (11.52–12.00)	11.58 (11.44–11.92)	0.10
CGI-58	6.67 (6.29–8.09)	7.58 (6.16–8.41)	0.09
Glucocorticoid receptor	9.62 (9.27–10.74)	10.49 (9.28–11.04)	0.05
Testosterone receptor	9.60 (9.21–10.09)	9.96 (9.37–10.59)	0.0004
Estrogen alpha receptor	6.26 (5.72–7.00)	6.72 (5.82–7.64)	0.0087
Estrogen beta receptor	5.30 (4.81–5.92)	5.38 (4.71–6.18)	0.61

Subjects with obesity were examined. Values are expressed as median and 25th quartile range. They were compared by Wilcoxon’s two sample test. A Bonnferroni corrected *p* value of <0.0018 was used as being truly statistically significant (see legend to Table 1 for details) and marked with bald style. *n* = sample size.

## Discussion

This study sheds new light on the nature of sex differences in adipose insulin resistance. Increased resistance among men is found when obesity is concomitantly present.

In participants without obesity, no sex difference in AdipoIR were observed although men without obesity had slightly but significantly higher values for HOMA-IR than women without obesity (Table S1). This may suggest organ specific effects on sex differences in insulin action because AdipoIR reflects adipose tissue and HOMA-IR liver and to some extent also skeletal muscle [31]. Discrepant results with comparisons of AdipoIR and HOMA-IR have been presented before as exemplified [32, 33].

When obesity was present the values for AdipoIR and HOMA-IR were much higher in men than women. Furthermore, lipolysis but not lipogenesis was subjected to a sex difference including differences between the sexes in the relationship to body fat. In subcutaneous fat cells insulin inhibition of lipolysis occurs at considerably lower concentrations than stimulation of lipogenesis [17]. It is therefore possible that lipolysis may be the most sensitive event of adipose tissue metabolism for a sex influence on insulin action. However, quantitatively, adipose tissue is likely less important than the other insulin target tissues for glucose utilization [34, 35].

The concentration-response experiments with fat cells made it possible to get some mechanistic insight regarding the sex differences in antilipolysis. Broadly, pD2 reflects insulin action at or near the receptor whereas responsiveness (maximum effect) mirrors distal events in hormone action [21]. Although responsiveness of antilipolysis was slightly higher in women than men the major sex difference was a 10-fold increased sensitivity among the women. This suggests that sex differences above all occur at early signal steps for insulin action.

In published reports on pan genomic gene expression in subcutaneous adipose tissue relatively few genes were subjected to a sex difference in their adipose expression [36, 37]. The sex dimorphism was found for genes in pathways regulating inflammation, adipogenesis and mitochondrial function. In the present targeted analysis of the canonical insulin signal pathway in obesity with RNAseq only one gene, *IRS1*, displayed statistically significant sex difference showing decreased expression in men. Furthermore, there was a large, 60%, difference between the sexes using a quantitative measurement of IRS-1 mRNA levels. Our findings may suggest an important role of IRS-1 for sex differences in adipose insulin resistance among those with obesity. The idea must, however, be further supported by other features of IRS-1 such as protein levels and phosphorylation status. Finally, indirect effects of sex variations in local inflammation, adipogenesis and mitochondrial function revealed by the pan genomic studies mentioned above could also be important for sex differences in adipose insulin resistance [36, 37].

The sex effect on AdipoIR and antilipolysis was independent of age, cardiometabolic disorder, BMI, nicotine use or physical activity status. Adipose tissue factors such as increased basal lipolysis rate may also influence insulin action in fat cells as discussed [38]. Indeed, we found that this lipolytic rate was about two times faster in men than women with obesity.

Among genes that may regulate basal lipolysis and indirectly, insulin action in fat cells, only *CIDEA*, *PDE3B* and receptors for testosterone displayed different expression in men with obesity (higher values) compared with women with obesity. These dissimilarities do not readily explain the metabolic data. CIDEA depletion increases basal lipolysis in human fat cells [39] and sex hormones protect from insulin resistance [40, 41]. PDE3B enzyme activity is inversely related to basal rate of lipolysis in abdominal human subcutaneous adipose tissue [42]. It is therefore possible that events not directly related to the expression of these genes are involved in the sex differences or that other regulatory factors of basal lipolysis recently reviewed [43] but not examined herein are important.

We propose the following model for sex differences in insulin action on adipose tissue. In obesity men are more insulin resistant than women owing to a less efficient inhibition of fat cell lipolysis. This dimorphism is attributed to hormone action at early step(s) is the insulin canonical signal pathway and involves *IRS1*. Increased rate of basal lipolysis in men with obesity may also explain why their adipose tissue is more resistant to insulin than in women. Adipose insulin resistance can be modified by pharmacotherapy [19] and life-style intervention [44]. Such treatments may reduce the risk of future glucose intolerance and T2DM in men because AdipoIR is an independent risk factor for future dysglycemia [45]. However, our conclusions are drawn from a cross-sectional investigation and need to be supported by prospective studies.

The present study has some limitations. We only investigated abdominal subcutaneous adipose tissue and depot differences in adipose tissue function are well documented [5, 46]. Unfortunately, it was not possible for ethical and practical reasons to simultaneously investigate several superficial and deep adipose regions in this type of investigation. In both the KAROLINSKA and DiOGenes cohorts more women than men were included. The sex difference in recruitment may partly be explained by the lower concern of men for obesity related health issues than women [47]. The study was not population-based. On the other hand, studies involving biopsies may never be random because of the invasive nature of the examinations. We did not investigate menstrual status, but insulin resistance is associated with hyperandrogenemia rather than menstrual irregularity [48].

In summary, in obesity adipose insulin resistance is more prominent in men than women. This is selective for the antilipolytic effect of the hormone in fat cells and may, at least in part, be linked the canonical insulin signaling pathway, particularly IRS-1, and increased basal rate of lipolysis.

### Supplementary information

Supplemental Table 1.

Supplemental Figure 1

Supplementary Figure and Table legends

Supplemental Table 2.

## Data Availability

The datasets generated and analyzed during the current study are available from the corresponding author on reasonable request.
