# Supplementary material for: Sex differences in adipose insulin resistance are linked to obesity, lipolysis and insulin receptor substrate 1
Source: Int J Obes (Lond). 2024 Mar 15;48(7):934–40. doi: 10.1038/s41366-024-01501-x (PMC11217000; doi:10.1038/s41366-024-01501-x)
Supplement: Supplementary file 1 — Supplemental Table 1. [file 41366_2024_1501_MOESM1_ESM.docx]

| Phenotype | KAROLINSKA | | | | | | DIOGENES | | |
| --- | --- | --- | --- | --- | --- | --- | --- | --- | --- |
|  | No obesity | | | Obesity | | |  |  |  |
|  | Females | Males | p-value | Females | Males | p-value | Females | Males | p-value |
| Number | 804 | 249 | - | 1539 | 538 | - | 234 | 115 | - |
| Age (years) | 45 (37- 50) | 50 (32 – 59) | 0.002 | 43 (33 – 52) | 48 (39 – 56) | <0.0001 | 40 (37 – 45) | 42 (38 – 45) | 0.03 |
| Body mass index (kg/m^2^) | 24 (22 – 26) | 25 (23 – 27) | <0.0001 | 39 (36 – 43) | 39 (36 – 44) | 0.13 | 34 (32 – 37) | 34 (32 – 38) | 0.33 |
| Body fat (%) | 33 (28 – 38) | 22 (17 – 25) | <0.0001 | 48 (44 – 50) | 35 (31 – 38) | <0.0001 | 45 (42 – 48) | 33 (30 – 37) | <0.0001 |
| fP-triglycerides  (mmol/l) | 0.8 (0.6 – 1.1) | 0.9 (0.7 – 1.3) | 0.0004 | 1.4 (1.1 – 2.0) | 1.8 (1.3 – 2.5) | <0.0001 | 1.1 (0.8 – 1.5) | 1.4 (1.1 – 1.9) | <0.0001 |
| fP-total cholesterol  (mmol/l) | 4.8 (4.2 – 5.6) | 4.8 (4.1 – 5.4) | 0.29 | 5.0 (4.4 – 5.8) | 5.1 (4.5 – 5.7) | 0.47 | 4.8 (4.2 – 5.5) | 5.2 (4.5 – 5.9) | 0.005 |
| fP-HDL cholesterol  (mmol/l) | 1.6 (1.3 – 1.9) | 1.3 (1.1 – 1.6) | <0.0001 | 1.2 (1.0 – 1.4) | 1.1 (0.9 – 1.3) | <0.0001 | 1.3 (1.1 – 1.5) | 1.1 (0.9 – 1.3) | <0.0001 |
| fP-glucose (mmol/l) | 4.9 (4.6 – 5.2) | 5.2 (4.8 – 5.8) | <0.0001 | 5.3 (5.0 – 5.9) | 5.8 (5.3 – 7.0) | <0.0001 | 5.0 (4.8 – 5.3) | 5.2 (4.9 – 5.5) | 0.0004 |
| fS-insulin (mU/l) | 5.4 (4.2 – 7.0) | 5.6 (4.2 – 8.0) | 0.14 | 13.9 (9.6 - 20.6) | 22.1 (13.7 – 32.7) | <0.0001 | 8.8 (5.9 – 13.1) | 11.9 (7.9 –17.3) | 0.0003 |
| fP-free fatty acids (mmol/l) | 0.58 (0.45-0.74) | 0.52 (0.39-0.66) | <0.0001 | 0.72 (0.56-0.87) | 0.65 (0.53-0.81) | <0.0001 |  |  |  |
| HOMA-IR (units) | 1.2 (0.9 – 1.6) | 1.3 (0.9 – 2.1) | 0.012 | 3.3 (2.2 – 5.3) | 5.9 (3.6 – 10.2) | <0.0001 | 2.0 (1.3 – 3.1) | 2.7 (1.9 – 4.2) | <0.0001 |
| Sedentary/active | 333/61 | 169/19 | 0.09 | 455/536 | 129/222 | 0.003 |  |  |  |
| Nicotine use (yes/no) | 178/369 | 56/190 | 0.003 | 391/891 | 124/330 | 0.22 |  |  |  |
| Cardiometabolic disease (yes/no) | 57/747 | 58/191 | <0.0001 | 383/1156 | 262/276 | <0.0001 |  |  |  |
