# Supplementary figures and images for: Sex differences in adipose insulin resistance are linked to obesity, lipolysis and insulin receptor substrate 1
Source: Int J Obes (Lond). 2024 Mar 15;48(7):934–40. doi: 10.1038/s41366-024-01501-x (PMC11217000; doi:10.1038/s41366-024-01501-x)

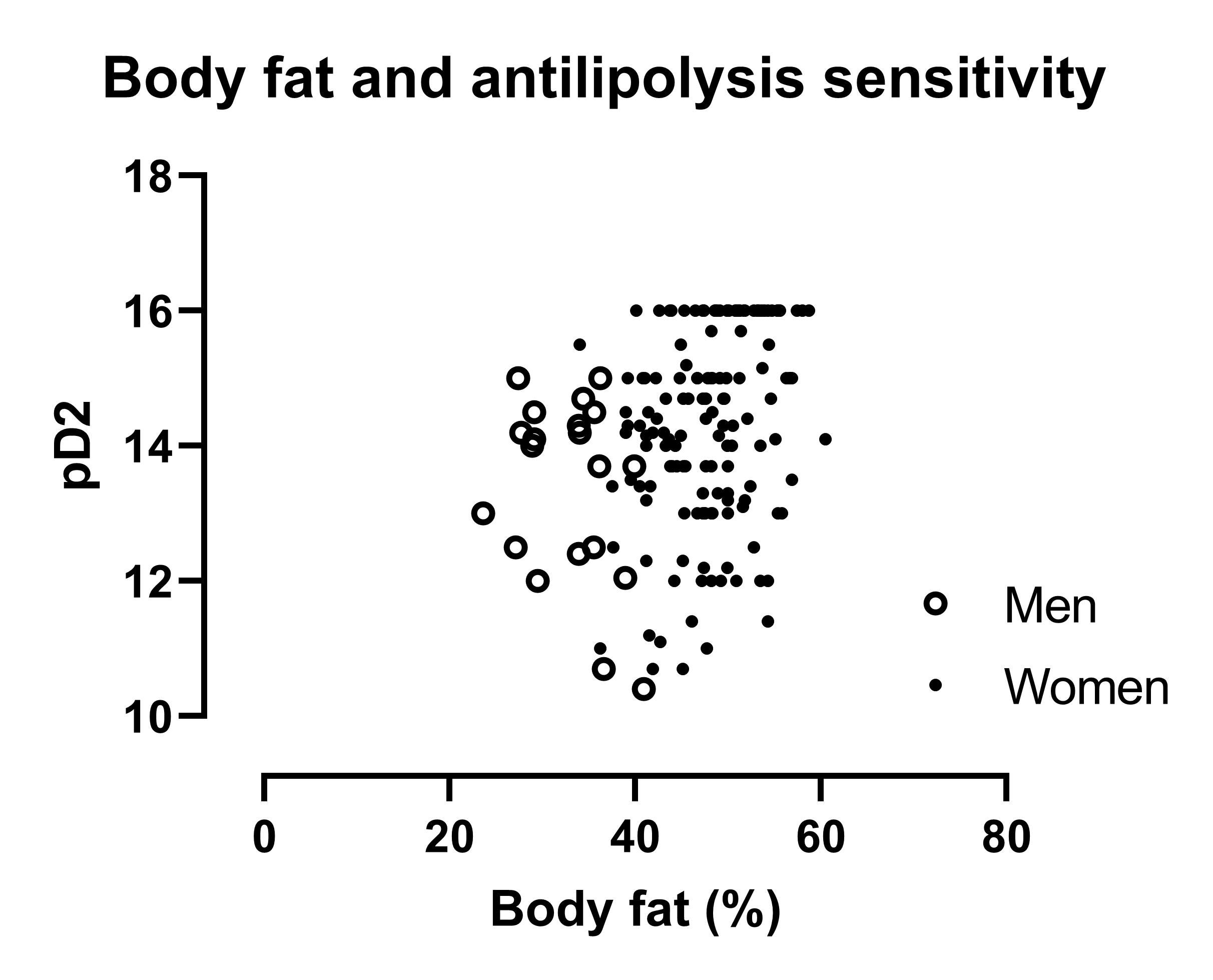

Supplement: Supplementary file 2 — Supplemental Figure 1 [file 41366_2024_1501_MOESM2_ESM.jpg]
