# Supplementary material for: Sex differences in adipose insulin resistance are linked to obesity, lipolysis and insulin receptor substrate 1
Source: Int J Obes (Lond). 2024 Mar 15;48(7):934–40. doi: 10.1038/s41366-024-01501-x (PMC11217000; doi:10.1038/s41366-024-01501-x)
Supplement: Supplementary file 3 — Supplementary Figure and Table legends [file 41366_2024_1501_MOESM3_ESM.docx]

Supplementary figure and table legends

*Figure S1.*

Relationship between percentage body fat and sensitivity of the antilipolytic effect of insulin (pD2) in obesity. Results were analyzed with Spearman correlation. Interaction with sex was examined by analysis of co-variance. Men are open circles and women are filled circles.

*Table S1.*

Clinical data. Values are median and (interquartile range). They were compared by Wilcoxon´s two sample test or Fisher’s exact test. fP or fS= fasting plasma or serum. The dichotomous phenotypes were not available in the DIOGENES group. Obesity is body mass index 30 kg/m^2^ or more and present in all DIOGENES subjects. Cardiometabolic disease is presence of diagnosed type 2 diabetes, hypertension, hyperlipidemia, or cardiovascular disease.
