# Supplementary material for: Sex differences in adipose insulin resistance are linked to obesity, lipolysis and insulin receptor substrate 1
Source: Int J Obes (Lond). 2024 Mar 15;48(7):934–40. doi: 10.1038/s41366-024-01501-x (PMC11217000; doi:10.1038/s41366-024-01501-x)
Supplement: Supplementary file 4 — Supplemental Table 2. [file 41366_2024_1501_MOESM4_ESM.docx]

| Model | Phenotype | AdipoIR | | pD2 antilipolysis | | Maximum antilipolysis | | *IRS-1* gene expression | |
| --- | --- | --- | --- | --- | --- | --- | --- | --- | --- |
|  |  | F-value | p-value | F-value | p-value | F-value | p-value | F-value | p-value |
| One | Sex | 34 | <0.0001 | 17 | <0.0001 | 9.6 | 0.0022 | 13.2 | 0.0003 |
|  | Age | 1.2 | 0.28 | 3.6 | 0.06 | 1.8 | 0.18 | 2.4 | 0.12 |
|  | Fasting glucose | 269 | <0.0001 | 10.7 | 0.0012 | 1.5 | 0.22 | 0.30 | 0.59 |
|  | Body mass index | 197 | <0.0001 | 0.03 | 0.87 | 0.05 | 0.83 | 4.9 | 0.027 |
| Two | Sex | 81 | <0.0001 | 14.4 | 0.0002 | 3.1 | 0.078 | 4.1 | 0.045 |
|  | Age | 1.3 | 0.26 | 3.0 | 0.086 | 1.6 | 0.21 | 1.7 | 0.25 |
|  | Fasting glucose | 187 | <0.0001 | 9.3 | 0.0025 | 0.02 | 0.88 | 0.12 | 0.74 |
|  | % body fat | 58 | <0.0001 | 2.5 | 0.11 | 0.04 | 0.84 | 0.7 | 0.39 |

Table S2. Influence of sex and co-factors on insulin action parameters in subcutaneous fat cells or adipose tissue from subjects with obesity. Two models were used differing in measure of body composition (body mass index in model one or % body fat in model two). Results were investigated by analysis of co-variance.
